# The Wide and Unpredictable Scope of Synthetic Cannabinoids Toxicity

**DOI:** 10.1155/2015/542490

**Published:** 2015-12-14

**Authors:** Jose Orsini, Christa Blaak, Eric Tam, Salil Rajayer, Joaquin Morante, Angela Yeh, Ashvin Butala

**Affiliations:** Department of Medicine, New York University School of Medicine, Woodhull Medical and Mental Health Center, 760 Broadway, Brooklyn, NY 11206, USA

## Abstract

Drug use and abuse continue to be a large public health concern worldwide. Over the past decade, novel or atypical drugs have emerged and become increasingly popular. In the recent past, compounds similar to tetrahydrocannabinoid (THC), the active ingredient of marijuana, have been synthetically produced and offered commercially as legal substances. Since the initial communications of their abuse in 2008, few case reports have been published illustrating the misuse of these substances with signs and symptoms of intoxication. Even though synthetic cannabinoids have been restricted, they are still readily available across USA and their use has been dramatically increasing, with a concomitant increment in reports to poison control centers and emergency department (ED) visits. We describe a case of acute hypoxemic/hypercapnic respiratory failure as a consequence of acute congestive heart failure (CHF) developed from myocardial stunning resulting from a non-ST-segment elevation myocardial infarction (MI) following the consumption of synthetic cannabinoids.

## 1. Introduction

Synthetic cannabinoids are a heterogeneous group of compounds developed to investigate possible therapeutic effects and to study endocannabinoid receptor systems. Clandestine laboratories subsequently utilized published data to developed synthetic cannabinoids variations marketed as designer drugs, which have been emerging as popular recreational drugs due to their easy accessibility and undetectability on standard toxicology screens. First sold under the name “Spice,” the drug has evolved into many different names (e.g., Black Diamond, Mojo, Spice Gold, Aroma, Dream, Genie, and Silver). K2 or “Spice” is made of a C8 homolog of the nonclassical cannabinoid CP-47, 497, 497-C8 (cannabicyclohexanol) and a cannabimimetic aminoalkylindole called JWH-018 [1]. They were distributed and sold legally in local smoke shops and gas stations in the USA until November 2010, when they were classified by the USA Drug Enforcement Agency (DEA) as Schedule-I controlled substances. Unlike partial agonist THC molecules, synthetic cannabinoids act as full nonselective agonists of the CB-1 and CB-2 receptors, making the substance 2–100 times more potent and longer lasting than THC [2]. They achieve euphoric effects by inhibiting glutamate synthesis and neurotransmission in the hippocampus [3].

Case reports of synthetic cannabinoids abuse have described patients presenting with alterations in mood and perception [4], xerostomia [5], and tachycardia [6]. Less common signs of intoxication included hypertension, agitation, paranoia, and hypokalemia [6]. There have been few cases describing more drastic features of intoxication such as ST-segment elevation MI [7], recurrent seizures [8], acute kidney injury [9], self-mutilation [10], serotonin syndrome [11], and cardiac arrest [12]. It has been estimated that more than 11,000 patients per year consult ED services in USA because of the side effects of synthetic cannabinoids [13]. As of May 2015, more than 40 deaths related to the use and abuse of synthetic cannabinoids have been reported in USA [14].

We describe a patient with acute hypoxemic/hypercapnic respiratory failure resulting from acute CHF developed from myocardial stunning as a consequence of a non-ST-segment elevation MI after consumption of synthetic cannabinoids.

## 2. Case Report

**Figure 1:**
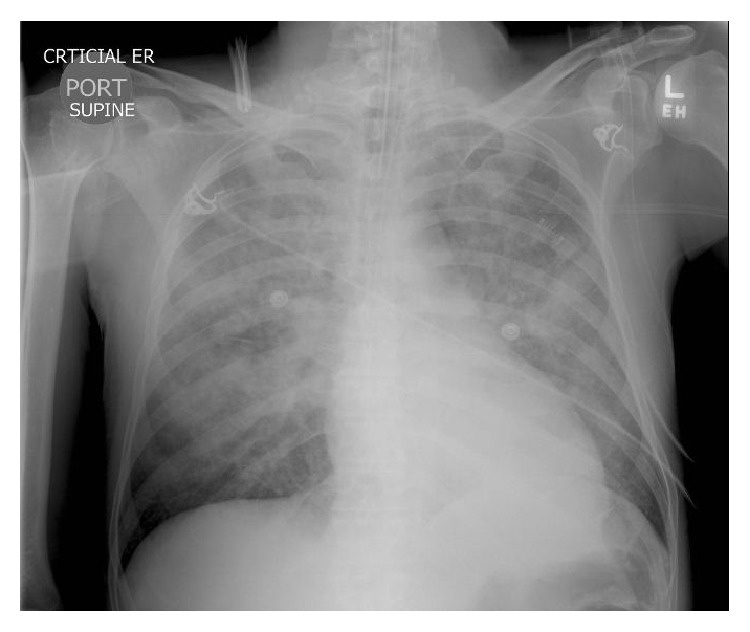
Initial CXR showing extensive bilateral infiltrates.

**Figure 2:**
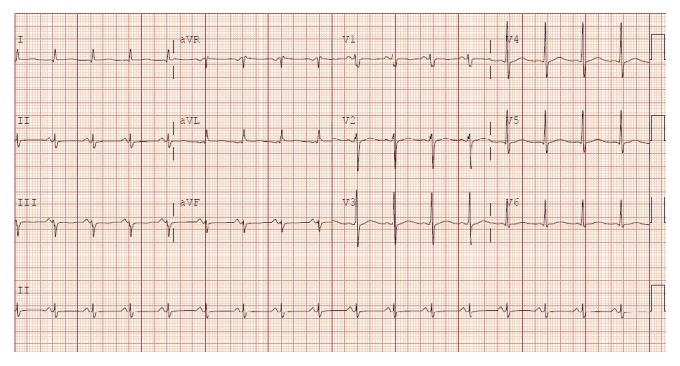
ECG showing sinus tachycardia without ST-segment or T-wave abnormalities.

A 41-year-old Hispanic male was brought to our hospital ED after having a witnessed tonic-clonic seizure on the street. His past medical history was significant for polysubstance abuse (heroin, cocaine, benzodiazepines, and methadone) and chronic liver disease (hepatitis C). He had history of multiple admissions to our institution for opiates detoxification. On arrival to ED his vital signs were as follows: blood pressure of 98/63 mmHg, heart rate of 118 beats/minute, respiratory rate of 48 breaths/minute, temperature of 37.5°C, and an oxygen saturation of 90% while receiving oxygen by a non-rebreather mask. Emergency Medical Services (EMS) staffing reported finding a bag of K2 at the scene. Physical examination was remarkable for bilateral rales on lung auscultation. Old needle puncture areas were found over his arms and legs, without erythema. Pupils were equal with positive light reflex. While in ED, he was combative and developed another tonic-clonic seizure episode. Endotracheal intubation was performed and he was placed on mechanical ventilation. Remarkable laboratory findings included a white blood cell (WBC) count of 29.5 K/mm^3^ (4.8–10.8), a bicarbonate level of 15 mmol/L (24–31), a lactic acid level of 4.4 mmol/L (0.5–2.2), and a urine toxicology screen positive for opiates, benzodiazepines, and methadone. Creatine kinase (CK) level was 5,590 U/L (25–215), and troponins were mildly elevated at 1.45 ng/mL (<0.1). Creatinine, coagulation, and liver function profiles were within normal limits. Arterial blood gas (ABG) while on mechanical ventilation and receiving FIO_2_ of 100% showed a pH of 7.14 (7.35–7.45), a pCO_2_ level of 79 mmHg (34–45), and a paO_2_ level of 77 mmHg (80–100). Initial chest X-ray (CXR) showed bilateral infiltrates (Figure 1). Electrocardiogram (ECG) showed sinus tachycardia without ST-segment or T-wave abnormalities (Figure 2).

**Figure 3:**
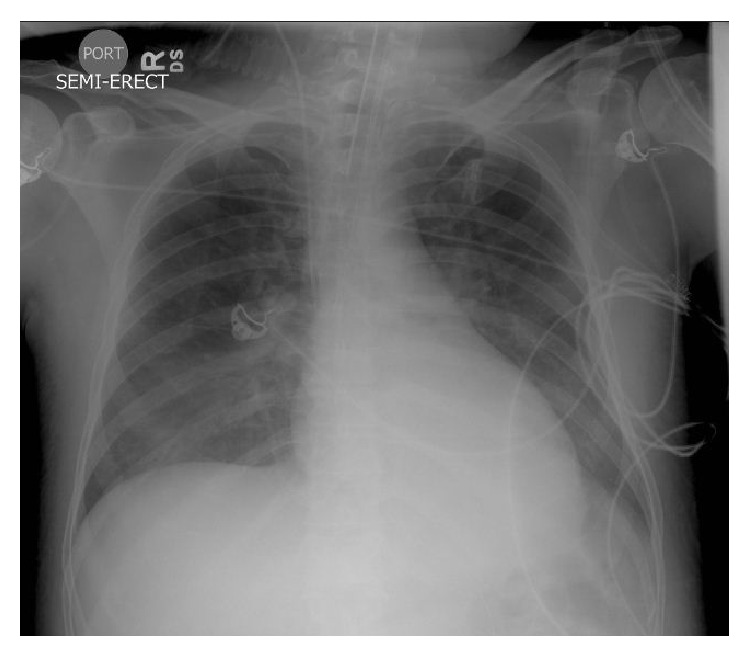
Follow-up CXR showing near resolution of bilateral infiltrates.

He was admitted to the intensive care unit (ICU) with the diagnosis of acute hypoxemic/hypercapnic respiratory failure presumptively secondary to drug overdose. He required propofol, fentanyl, and midazolam to achieve adequate sedation and ventilatory synchrony. Empiric intravenous antimicrobial therapy consisting of piperacillin/tazobactam (3.375 grams every 6 hours) was initiated for the possibility of aspiration pneumonitis. While in ICU, CK and troponin levels continued to increase to 18,589 U/L and 8.76 ng/mL, respectively. A new transthoracic echocardiogram (TTE) showed markedly decreased left ventricular ejection fraction of 30% (55–65), with severe global and segmental hypokinesis and no vegetations or valvular dysfunction. Given the echocardiographic and CXR findings as well as the elevated troponin levels, therapy for acute congestive heart failure probably secondary to a non-ST-segment elevation MI was initiated with low-molecular weight heparin, *β*-blockers, aspirin, clopidogrel, diuretics, statins, and angiotensin-converting enzyme (ACE) inhibitors. Repeated CXR after 24 hours showed near resolution of bilateral infiltrates (Figure 3). Blood, urine, and respiratory cultures were negative and antimicrobials were discontinued. He was extubated on day 4 of ICU admission but required reintubation because of severe agitation and hypoxemia, which were thought to be a clinical component of a possible withdrawal syndrome. His ICU course was further complicated by fevers, new bilateral infiltrates on CXR, and persistently elevated FIO_2_ requirements. TTE was repeated, showing a remarkable improvement of left ventricular ejection fraction (63%), with a complete resolution of wall motion abnormalities. He was successfully extubated on day 11 of ICU admission, after being treated for acute respiratory distress syndrome (ARDS) secondary to ventilator-associated pneumonia.

## 3. Discussion

It is known that marijuana has pathophysiological effects on the cardiovascular system, which are mediated by stimulation of the sympathetic nervous system through release of norepinephrine and by parasympathetic blockade [15]. Marijuana consumption increases oxygen demands on the myocardium and also leads to an increase in carboxyhemoglobin levels, which results in decreased oxygen-carrying capacity [16, 17]. Interference with the integrity of peripheral vascular response has been postulated to be one of the mechanisms for cardiac events during cannabis smoking [14]. THC may also be associated with vascular inflammation and increased platelet activation, which is a potential mechanism of plaque rupture [18]. Few cases of MI associated with marijuana use have been reported in the literature [19–22]. Myocardial ischemia has also been reported with the use of synthetic cannabinoids [7, 23, 24]. In a large epidemiological study, THC and derivatives were reported to increase the risk of MI by 4.8 times in the first hour after use [25]. Given the presence of other substances in the urine toxicology screen which have been linked to acute coronary events, it was challenging to categorize the etiology of our patient's cardiovascular findings. Information about heroin-related MI is limited and its mechanisms are not well established. It has been postulated that heroin might have a direct toxic effect on the coronary arteries leading to coronary occlusion, either by provoking a local coronary spasm or inflammation [26]. The hypothesis of heroin-related myocardial injury might include rhabdomyolysis with cardiac involvement, hypoxia, acidosis, and vasoconstrictive substances released by muscle necrosis [27]. This hypothesis may explain the findings of rhabdomyolysis, hypoxia, and acidosis on the patient described in this report. However, the fact that our patient's pupils were not constricted and a bag of K2 was found at the scene makes heroin a less likely etiology for this patient's cardiac abnormalities. Although cases of myocardial ischemia possibly related to methadone use have been reported [28, 29], it has been proposed that methadone possesses cardioprotective properties that include reduction in infarct size in patients with myocardial infarction [30]. Cocaine was not found in the urine toxicology screen in our patient, which makes that substance an extremely unlikely cause for this patient's cardiovascular derangements.

To the best of our knowledge, this is the first report of synthetic cannabinoid-induced non-ST-segment elevation MI resulting in myocardial stunning and acute CHF in a patient without proven history of coronary artery disease. We hypothesized that our patient likely had transient myocardial ischemia resulting in ventricular stunning that might had led to acute CHF. This hypothesis is supported by the fact that subsequent troponin levels normalized within 48 hours and repeated TTE showed complete resolution of wall motion abnormalities with normal left ventricular ejection fraction. Patients with non-ST-segment elevation MI may present with heterogeneous conditions and, therefore, they may have varying degrees of reduction of coronary blood flow but without complete coronary occlusion in combination with distal embolization of thrombotic material and accompanying coronary spasm. In addition, myocardial necrosis (expressed by troponin elevation) may occur in the absence of coronary thrombosis but in the presence of stable but diffuse coronary artery disease and clinical conditions that increase myocardial demands (type-2 MI). Although the most common ECG findings in patients with non-ST-segment elevation MI are ST-segment depression and T-wave inversion, the presence of a normal ECG does not exclude the diagnosis of non-ST-segment elevation MI. Some studies have shown that approximately 1%–6% of patients with normal ECGs are found to have either an acute MI or unstable angina [31, 32].

Even though the main limitation of this report is the lack of biological testing of the patient's blood specimen, our subject's toxidrome fits perfectly with K2 overdose. Agitation and tachycardia are known side effects of synthetic cannabinoids [6]. Seizures have been reported in patients with synthetic cannabinoids intoxication [8, 33]. Rhabdomyolysis has been described in individuals abusing synthetic cannabinoids [9, 34]. Surely, the tonic-clonic seizures our patient developed contributed to the elevated CK levels. It is possible that myositis secondary to an autoimmune reaction to some of the inhaled antigens contained in synthetic cannabinoids may have played a role in the etiology of our patient's rhabdomyolysis. Another potential limitation of this paper is the lack of further follow-up studies such as coronary angiography and cardiac magnetic resonance imaging.

Our patient displayed other interesting clinical findings not commonly described in the literature. Based on a MEDLINE search and using the words “hypoxemia”, “hypercapnea”, “respiratory failure”, and “synthetic cannabinoids”, hypoxemic and/or hypercapnic respiratory failure has been infrequently reported in association with the consumption of synthetic cannabinoids. Berkowitz et al. reported a case series of patients with hypoxemic respiratory failure following the inhalation of synthetic cannabinoids [35]. Similarly, Alhadi et al. described another case of hypoxemia after the use of cannabinoids [36]. Hypoxemia related to synthetic cannabinoids use has also been outlined by Aksel et al. [37]. Diffuse miliary-micronodular infiltrates and centrilobular nodules with tree-in-bud pattern are the most common radiologic findings in patients with respiratory failure resulting from the use of synthetic cannabinoids [35]. The effects of synthetic cannabinoids on respiratory function have not been extensively detailed in humans and likely involve multiple mechanisms. Studies in rats demonstrated a marked respiratory depression, characterized by a decrease in respiratory rate, hypoxia, hypercapnea, and acidosis. Synthetic cannabinoids effect on peripheral receptors, such as chemo- and baroreceptors, increased airway resistance in bronchi, making CB-1 receptors stimulation a possible hypothesis for synthetic cannabinoid-induced respiratory depression [38]. Chemical gases released after inhalation of these substances may also cause damage to the bronchiolar epithelium, leading to acute respiratory distress that may progress to respiratory failure. We hypothesized that the etiology of respiratory failure in our patient was most likely acute congestive heart failure triggered by synthetic cannabinoids overdose.

## 4. Conclusion

This case illustrates to health care workers the possible life-threatening adverse effects of synthetic cannabinoids abuse. Although there is no specific toxidrome associated with synthetic cannabinoids intoxication, clinicians should suspect their involvement in patients presenting with signs and symptoms of drug overdose. Health care providers, especially those working in ED and critical care settings, should be on alert for drug-induced toxicities. Further research is needed to identify which contaminants are usually found in synthetic cannabinoids and to understand the interaction between different types of these substances to better predict adverse outcomes.
